# Draft Genome Sequence of *Streptomyces canus* ATCC 12647, a Producer of Telomycin

**DOI:** 10.1128/genomeA.00173-16

**Published:** 2016-03-24

**Authors:** Dennis Y. Liu, Yongchang Li, Nathan A. Magarvey

**Affiliations:** Department of Biochemistry and Biomedical Sciences, Department of Chemistry and Chemical Biology, McMaster University, Hamilton, Ontario, Canada

## Abstract

We present here the genome sequence of *Streptomyces canus* ATCC 12647, a producer of the antibiotic telomycin, noted for its unique antibacterial activity against cardiolipin. Genomic analysis using the bioinformatics tool PRISM revealed the presence of multiple biosynthetic gene clusters, including those for telomycin and other natural products of potential pharmacological interest.

## GENOME ANNOUNCEMENT

Soil environments have long been appreciated as an important source of microorganisms that produce diverse bioactive natural products. Of these organisms, *Streptomyces* spp. are well known for their plentiful biosynthetic gene clusters, which encode a wide array of small molecules produced through modular assembly line enzymes. *Streptomyces canus* C-509 (ATCC 12647) is a notable member of this group and was previously isolated by researchers of the Bristol-Myers Company (now Bristol-Myers Squibb Co.) in the 1950s, who identified its production of telomycin, an antibiotic with noteworthy bactericidal activity (1, 2). To investigate the biosynthetic origins of telomycin and any other natural products of potential interest, we sequenced the genome of *S. canus*.

Genomic DNA was obtained from the cultivation of *S. canus* ATCC 12647 (American Type Culture Collection) using a previously described protocol (3). Bacterial DNA was isolated using a GenElute bacterial genomic DNA kit (Sigma). Sequencing was performed by the Farncombe Metagenomics Facility at McMaster University on an Illumina HiSeq DNA sequencer using TruSeq Rapid version 1 chemistry with cBot hybridization. The associated library was prepared using a NEBNext DNA library preparation kit (New England BioLabs) and sequenced via a 2 × 151-bp reagent kit, providing approximately 14.5 million reads and 100× average coverage. Raw reads were assembled through Slapline using the SPAdes algorithm (4), followed by the Geneious assembler. The draft genome contains 7,794,673 nucleotides, with a G+C content of 72.0%. A total of 64 contigs were obtained, with Glimmer version 3.02b (5) identifying 7,360 putative protein-coding genes in 45 contigs.

The assembled genome was examined using PRISM 1.2.7 (6), and 12 secondary metabolite biosynthetic gene clusters were identified, of which 3 were nonribosomal peptides, 6 were polyketides, 1 was another hybrid peptide-polyketide, and 2 were other metabolites. From these results, we identified the complete nonribosomal peptide gene cluster responsible for the production of telomycin and its associated analogues. Genomic analysis of this cluster led to our isolation and characterization of telomycin’s unique chemical scaffold and activity against bacterial membrane cardiolipin. In addition to this established product, we also observed gene clusters with high homology scores corresponding to those known to produce coelichelin and albaflavenone. Other remaining nonribosomal peptide, polyketide, and hybrid clusters of substantial size are also present with yet-unknown identity.

Different strains of *S. canus* have been studied extensively in the past and have revealed a range of compounds with antifungal and bactericidal activities (7). Here, we sequenced the genome of *S. canus* ATCC 12647, a known producer of the cardiolipin-targeting antibiotic telomycin. Our analysis has led to the discovery of telomycin’s unique mode of action, as well as the presence of numerous other nonribosomal peptide and polyketide clusters of potentially interesting chemistry and activity within this organism.

### Nucleotide sequence accession numbers.

This whole-genome shotgun project has been deposited at DDBJ/EMBL/GenBank under the accession no. LQCG00000000. The version described in this paper is the first version, LQCG01000000.
